# H2A *O*-GlcNAcylation at serine 40 functions genomic protection in association with acetylated H2AZ or γH2AX

**DOI:** 10.1186/s13072-017-0157-x

**Published:** 2017-10-30

**Authors:** Koji Hayakawa, Mitsuko Hirosawa, Ruiko Tani, Chikako Yoneda, Satoshi Tanaka, Kunio Shiota

**Affiliations:** 1 Laboratory of Cellular Biochemistry, Department of Animal Resource Sciences/Veterinary Medical Sciences, The University of Tokyo, Tokyo, 113-8657 Japan; 2 Waseda Research Institute for Science and Engineering, Waseda University, Tokyo, 169-8555 Japan

**Keywords:** *O*-GlcNAcylation, Histone modification, Canonical histone, Histone variant, DNA damage, DNA repair

## Abstract

**Background:**

We have previously reported a novel *O*-GlcNAc modification at serine 40 (S40) of H2A (H2AS40Gc). S40-type H2A isoforms susceptible to *O*-GlcNAcylation are evolutionarily new and restricted to the viviparous animals; however, the biological function of H2AS40Gc is largely unknown. H2A isoforms are consisted of S40 and alanine 40 (A40) type and this residue on H2A is located in the L1 of the globular domain, which is also known as a variable portion that distinguishes between the canonical and non-canonical H2A variants. In this study, by considering the similarity between the S40-type H2A and histone H2A variants, we explored the function of H2AS40Gc in mouse embryonic stem cells (mESCs).

**Results:**

We found several similarities between the S40-type H2A isoforms and histone H2A variants such H2AZ and H2AX. mRNA of S40-type H2A isoforms (H2A1 N and H2A3) had a poly(A) tail and was produced throughout the cell cycle in contrast to that of A40-type. Importantly, H2AS40Gc level increased owing to chemical-induced DNA damage, similar to phosphorylated H2AX (γH2AX) and acetylated H2AZ (AcH2AZ). H2AS40Gc was accumulated at the restricted area (± 1.5 kb) of DNA damage sites induced by CRISPR/CAS9 system in contrast to accumulation of γH2AX, which was widely scattered. Overexpression of the wild-type (WT) H2A3, but not the S40 to A40 mutation (S40A-mutant), protected the mESC genome against chemical-induced DNA damage. Furthermore, 3 h after the DNA damage treatment, the genome was almost recovered in WT mESCs, whereas the damage advanced further in the S40A-mutant mESCs, suggesting functions of H2AS40Gc in the DNA repair mechanism. Furthermore, the S40A mutant prevented the accumulation of the DNA repair apparatus such as DNA-PKcs and Rad51 at the damage site. Co-immunoprecipitation experiment in WT and S40A-mutant mESCs revealed that H2AS40Gc physiologically bound to AcH2AZ at the initial phase upon DNA damage, followed by binding with γH2AX during the DNA damage repair process.

**Conclusions:**

These data suggest that H2AS40Gc functions to maintain genome integrity through the DNA repair mechanism in association with AcH2AZ and γH2AX.

**Electronic supplementary material:**

The online version of this article (doi:10.1186/s13072-017-0157-x) contains supplementary material, which is available to authorized users.

## Background

*O*-linked β-*N*-acetylglucosamine (*O*-GlcNAc) modifications occur at serine (Ser) or threonine (Thr) residues in a number of cytosolic and nuclear proteins including transcription, stem cell, and epigenetic factors as well as histones [1–5]. The Ser or Thr residue is also the site of phosphorylation in various proteins, and a role in the competition between *O*-GlcNAcylation and phosphorylation has been postulated [3]. To date, *O*-GlcNAcylation on various histones has been reported and the several biological activities have been postulated [6–12]. For example, *O*-GlcNAcylation at histone H2B serine 112 (H2BS112) is reportedly linked to transcriptional activation via the promotion of H2B ubiquitination [9].

We recently reported a novel *O*-GlcNAc modification at serine 40 (S40) of some H2A isoforms (H2AS40Gc) [13]. Distinct from other histone modifications, H2AS40Gc is restricted to viviparity-specific H2A isoforms. In mammals, there are multiple canonical histone H2A isoforms consisting of isoforms with S40 and alanine 40 (A40), whereas non-mammalian species harbor only A40-type H2A members. Thus, the A40 type seems to be a prototype of H2A, and S40-type H2A isoforms, which are susceptible to *O*-GlcNAcylation, are evolutionarily new. In our previous study, ChIP-seq analysis revealed that H2AS40Gc is predominantly distributed in genic regions and is associated with gene expression in mouse trophoblast stem cells [13]. However, the biological functions of H2AS40Gc are poorly understood.

Histones are classified into two types, canonical histones and histone variants (non-canonical histones), based on the similarity of the amino acid sequences and several other criteria [14–21]. Canonical histones are encoded as gene clusters and are synthesized at the S phase of the cell cycle [14, 16–19]. In contrast, each histone variant is encoded alone on other chromosomes and produced in a cell cycle-independent manner [20]. Moreover, mRNA of canonical histones has a unique 3′-stem–loop structure instead of a poly(A) tail, whereas mRNA of histone variants has a poly(A) tail [14, 20]. The functions of histone variants have been studied well, e.g., H2AX and H2AZ have a role in repairing damaged DNA [14, 21–29].

**Fig. 1:**
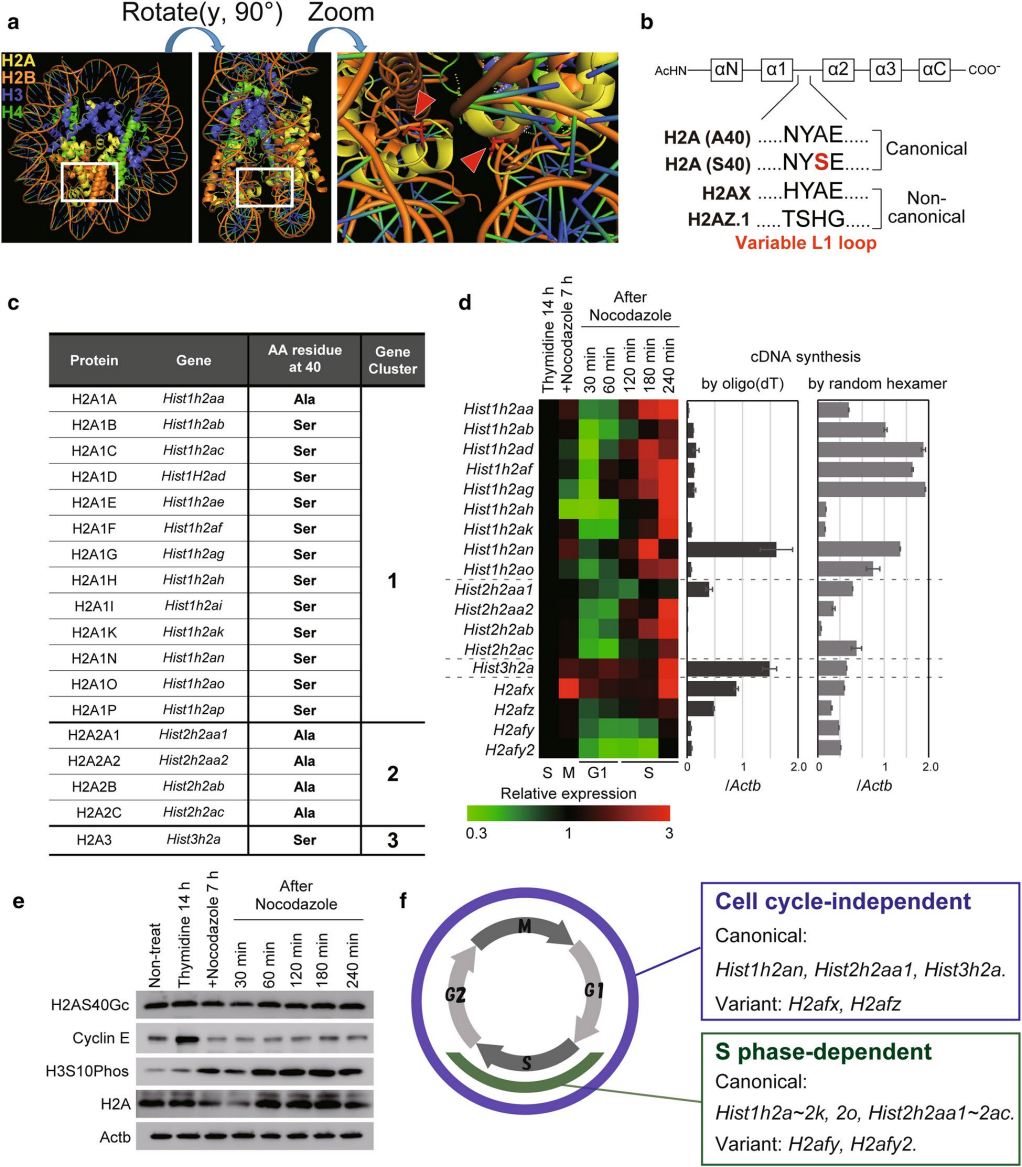
Cell cycle-independent H2AS40Gc production. **a** Three-dimensional reconstruction map of core histones. Arrowheads indicate residue 40 of H2A. **b** Sequence diversity of L1 loop region in H2A. **c** List of canonical H2As in mice. This list is based on Refseq database (mouse mm9). **d** Left, expression level of H2A-coding genes in synchronized mESCs. Values are expressed as relative to that of thymidine-treated (14 h) mESCs, which is set as 1. Right, expression level of mRNA for H2A-coding genes using cDNA synthesized using oligo(dT) primer or random hexamer (RH) in non-synchronized mESCs. Values are normalized by *Actb* expression. **e** H2AS40Gc level in synchronized mESCs. Cyclin E and phosphorylated H3S10 (H3S10Phos) were used to determine cell cycle phases. Actb was used as the internal control. **f** Summary of canonical and variant H2A gene expression through cell cycle

The site of *O*-GlcNAcylation at S40 of H2A is located in the L1 site of the globular domain where two histone H2A/H2B dimers associate with each other in the nucleosome (Fig. 1a) [16, 21, 30–32]. L1 is also referred to as a variable portion that distinguishes between canonical and non-canonical H2A variants such as H2AX and H2AZ (Fig. 1b) [16, 22]. The structure of L1 with A40/S40 or *O*-GlcNAcylated S40 may contribute to the production of a diverse nucleosome with H2A histone variants. Thus, discovering H2AS40Gc prompted us to explore whether the S40-type H2A isoforms are expressed as ordinary canonical histones or as unique histone-like variants and whether there are structural differences in the 3′ tail of the mRNA between S40- and A40-type H2A. Furthermore, by considering the analogy of S40-type H2A isoforms and histone variants, the function of H2AS40Gc can be studied. Here, we show that some genes for S40-type H2A, susceptible to *O*-GlcNAcylation, are expressed in a cell cycle-independent manner, similar to that of histone variants. In addition, we revealed that H2AS40Gc is responsive to DNA damage and plays a role in DNA damage repair (DDR) in association with phosphorylated H2AX (γH2AX) and acetylated H2AZ (AcH2AZ).

## Results

### Gene expression of H2AS40 isoforms in a cell cycle-independent manner

There are 18 canonical histone H2A isoforms, of which thirteen S40 and five A40 types are present in mouse (Fig. 1c). Seven S40 types and one A40 type are located on gene cluster 1, whereas no S40 types are located on cluster 2. Of note, the H2A3 gene (*Hist3h2a*) is solitary in the genome similar to histone variants.

We investigated the expression profile of 14 H2A isoforms, including S40 and A40 types, during cell cycle by RT-qPCR using specific primer sets (Additional file 1: Table S1) in mouse embryonic stem cells (mESCs) synchronized by combinatorial treatment with thymidine and nocodazole (Fig. 1d, *left*). RT-qPCR analysis revealed expression of the H2AX-encoding gene (*H2afx*) throughout the cell cycle, similar to a previous study [20]. The H2AZ-encoding gene (*H2afz*) also showed cell cycle-independent expression, whereas the expression of other variants (*H2afy* and *H2afy2*) decreased during the G1 phase.

Intriguingly, *Hist3h2a* (encoding the canonical histone H2A3) was constantly expressed throughout the cell cycle (Fig. 1d, *left*) similar to *H2afx*. Other members such as *Hist1h2an* (S40 type), *Hist2h2aa1* (A40 type) were also expressed regardless of the cell cycle. Remaining members of H2A were expressed in a typical S-phase-dependent pattern. Thus, some S40-type H2A isoforms were produced throughout the cell cycle. Western blotting analysis of synchronized mESCs showed that H2AS40Gc was constantly observed throughout the cell cycle in contrast to histone H3 phosphorylation at S10 (H3S10Phos; Fig. 1e).

To investigate the structure of the 3′ tail of H2A mRNA, we prepared cDNA synthesized by oligo(dT) primers or random hexamers. Almost all of the H2A members were highly expressed using the cDNA synthesized by random hexamers (Fig. 1d, *right*), while their expression levels were low or undetectable in the cDNA using oligo(dT) primers (Fig. 1d, *middle*). These data support the findings of previous reports indicating that genes encoding histone H2A that are primarily expressed at the S phase harbor the 3′-stem–loop structure instead of a poly(A) structure [14, 19, 20]. By contrast, RT-qPCR using the cDNA prepared by oligo(dT) primers confirmed that *H2afx* and *H2afz* mRNA had a poly(A) structure (Fig. 1d, *middle*). Interestingly, the mRNAs for *Hist1h2an*, *Hist2h2aa1*, and *Hist3h2a*, but not other mRNAs for remaining members, could be detected by the oligo(dT)-based cDNA.

Taken together, these data clarify that some H2A isoforms (*Hist1h2an, Hist2h2aa1,* and *Hist3h2a*) are expressed independent of the cell cycle and have a poly(A) structure similar to genes for H2A variants such as *H2afx* and *H2afz* (Fig. 1f). Among the thirteen S40-type H2A members, H2A3 and H2A1N are the only members that were expressed in a cell cycle-independent manner and their mRNA contained a poly(A) tail structure.

### DNA damage induced H2AS40Gc foci overlapping with γH2AX and AcH2AZ

We previously observed that H2AS40Gc can be recognized as dotted foci in the nucleus of mouse trophoblast stem cells, germ cells, and ESCs with immunofluorescence (IF) analysis [13]. Considering that the formation of dotted foci is a characteristic of γH2AX and AcH2AZ [16, 21–29], H2AS40Gc may have a similar function to H2AX and H2AZ. Since γH2AX and AcH2AZ are involved in maintaining genome integrity and the DDR process, we next investigated the response of H2AS40Gc against DNA damage.

**Fig. 2:**
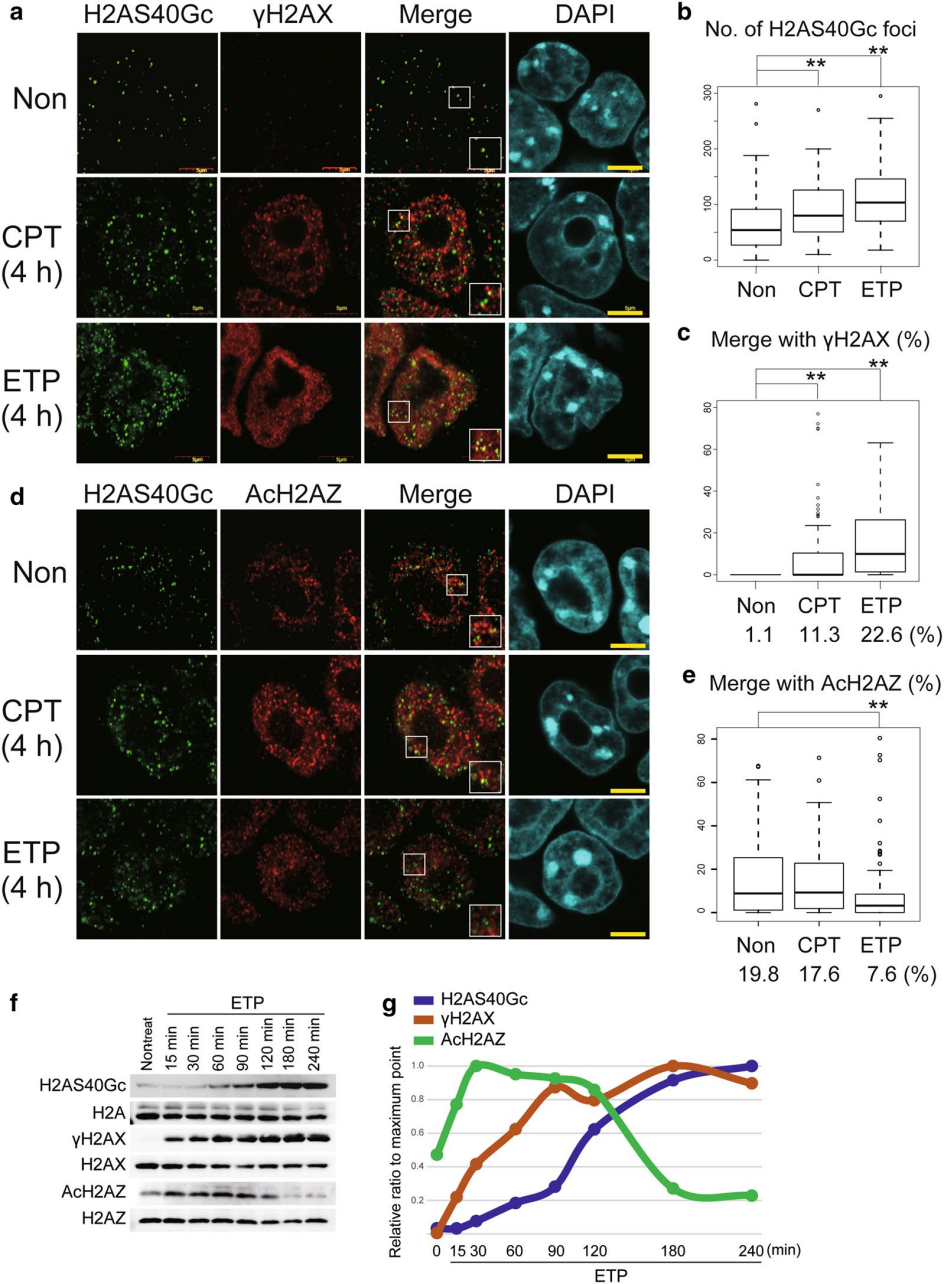
H2AS40Gc is responsive to DNA damages. **a**–**e** Immunofluorescence (IF) images of H2AS40Gc, γH2AX, and AcH2AZ in mESCs treated with camptothecin (CPT) and etoposide (ETP). The lower right box in the photo is the zoomed white box in the nucleus. Bars = 5 μm. Measurement of the number of H2AS40Gc foci (**b**) and co-localization of H2AS40Gc with γH2AX (**c**) or AcH2AZ (**e**) in nuclei of mESCs treated with CPT and ETP. **p < 0.01. **f**, **g** Time course of accumulation of H2AS40Gc, γH2AX, and AcH2AZ proteins in ETP-treated mESCs. The graph was plotted from band intensities of **f** calculated by the ImageJ software (**g**). Values of H2AS40Gc, γH2AX, and AcH2AZ were normalized to the intensity of α-H2A, -H2AX, and -H2AZ, respectively, and expressed as a ratio relative to the maximum

IF assay confirmed that H2AS40Gc was visualized as dotted foci in mESCs (Fig. 2a). When the cells were treated for 4 h with topoisomerase inhibitors, camptothecin (CPT) or etoposide (ETP), IF revealed an increase in the number of H2AS40Gc foci (Fig. 2a, b). In parallel, the number of γH2AX foci also increased following the topoisomerase inhibitor treatment (Fig. 2a). The overlapping of H2AS40Gc foci with γH2AX foci increased to 11.3 and 22.6% in CTP- and ETP-treated cells, respectively, compared to those in non-stimulated control cells (Fig. 2a, c).

More than 19% of the H2AS40Gc foci overlapped with AcH2AZ in non-treated mESCs (Fig. 2d, e). CTP and ETP treatments caused an increase in the AcH2AZ signals. The foci of H2AS40Gc overlapped with AcH2AZ foci at 17.6 and 7.6% in CTP and ETP treatments, respectively. Thus, overlapping of H2AS40Gc with AcH2AZ is reciprocal to that with γH2AX before and after the treatments. Western blotting analysis of nuclear fractions revealed that H2AS40Gc levels started increasing 60 min after the initiation of ETP treatment, whereas γH2AX levels increased after 15 min and then increased gradually (Fig. 2f, g). After 90 and 180 min of the treatment, levels of γH2AX and H2AS40Gc, respectively, reached at high levels (Fig. 2g). Nuclear accumulation of AcH2AZ was high 15 min after ETP treatment and reached the maximum level at 30 min. The high level of AcH2AZ was maintained until 90–120 min when H2AS40Gc accumulation started, and then reduced to the basal level after 120 min (Fig. 2g).

Taken together, this is the first report to demonstrate that induction of H2AS40Gc is responsive to the DNA damages. The nuclear accumulation of H2AS40Gc, γH2AX, and AcH2AZ and their co-localization exhibit dramatic changes during the DNA damage responses.

### Accumulation of H2AS40Gc at sites of DNA damage induced by CRISPR/CAS9 system

**Fig. 3:**
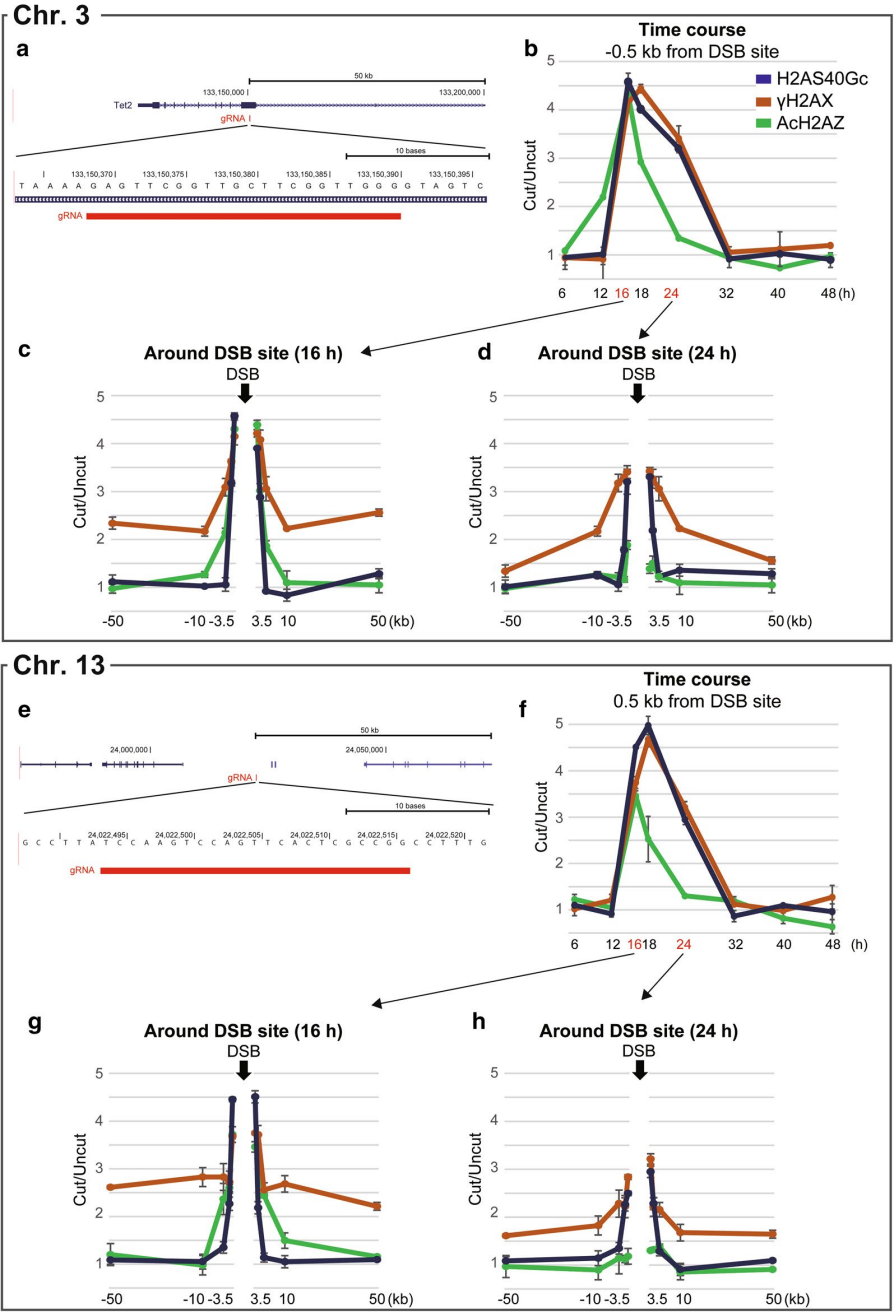
H2AS40Gc in sites of DNA damages.** a**–**h** Level of H2AS40Gc, γH2AX, and AcH2AZ around the damaged DNA site induced by the CRISPR/CAS9 system. gRNA-target regions were selected at gene-body (chromosome 3) (**a**) and intergenic region (chromosome 13) (**e**). Level of H2AS40Gc and γH2AX at − 0.5 kb from damaged DNA site on chromosome 3 (**b**) and 0.5 kb from the damaged DNA site on chromosome 13 (**f**). ChIP signals were calculated as ChIP DNA/Input DNA and expressed as fold enrichment relative to the uncut (mESCs expressing guide RNA and nuclease-deficient CAS9). Values indicate mean ± SD (*n* = 3). Enrichment of H2AS40Gc and γH2AX around the DNA damage site on chromosome 3 (**c**, **d**) and chromosome 13 (**g**, **h**) induced using CRISPR/CAS9, at 16 and 24 h after transfection. Values indicate mean ± SD (*n* = 3). DSB, double-strand DNA break

To confirm H2AS40Gc localization at the genomic damage loci, we induced locus-specific DNA damage by using CRISPR/CAS9 system, wherein the guide RNA (gRNA) was designed for the two sites on exon 9 of the *Tet2* gene on chromosome 3 (Fig. 3a) and the intergenic area on chromosome 13 (Fig. 3e). ChIP signals were calculated as ChIP DNA/input DNA and expressed as fold enrichment relative to the uncut (mESCs expressing guide RNA and nuclease-deficient CAS9). Using γH2AX as the DNA damage marker, the CRISPR/CAS9-induced genome damage on chromosomes 3 and 13 could be detected after 16 h and continued until 24 h after the transfection (Fig. 3b, f). The increase in H2AS40Gc localization was parallel to that of γH2AX in the time-course study.

At 16 h after transfection, distribution of γH2AX was confirmed in the relatively wide range of ± 50 kb around the DNA damage sites, as previously reported [24, 27], on both chromosomes 3 and 13 (Fig. 3c, g). H2AS40Gc was also observed at the damaged sites. Interestingly, however, H2AS40Gc localized within a narrow range within the ± 1.5 kb band from the DNA damage site. At 24 h after transfection, accumulation of H2AS40Gc was observed within the narrow area, whereas the occupancy band of γH2AX was more than ± 50 kb on the cutting sites (Fig. 3d, h). Disappearance of AcH2AZ occurred at the DNA damage sites earlier than the disappearance of H2AS40Gc and γH2AX at 24 h.

Thus, we confirmed the accumulation of H2AS40Gc at the DNA damage sites. Interestingly, H2AS40Gc did not expand its territory for the DDR process as observed in the case of γH2AX and was retained at the center of the DNA damage regardless of genic and intergenic regions.

### Genomic protection and DDR by H2AS40Gc

**Fig. 4:**
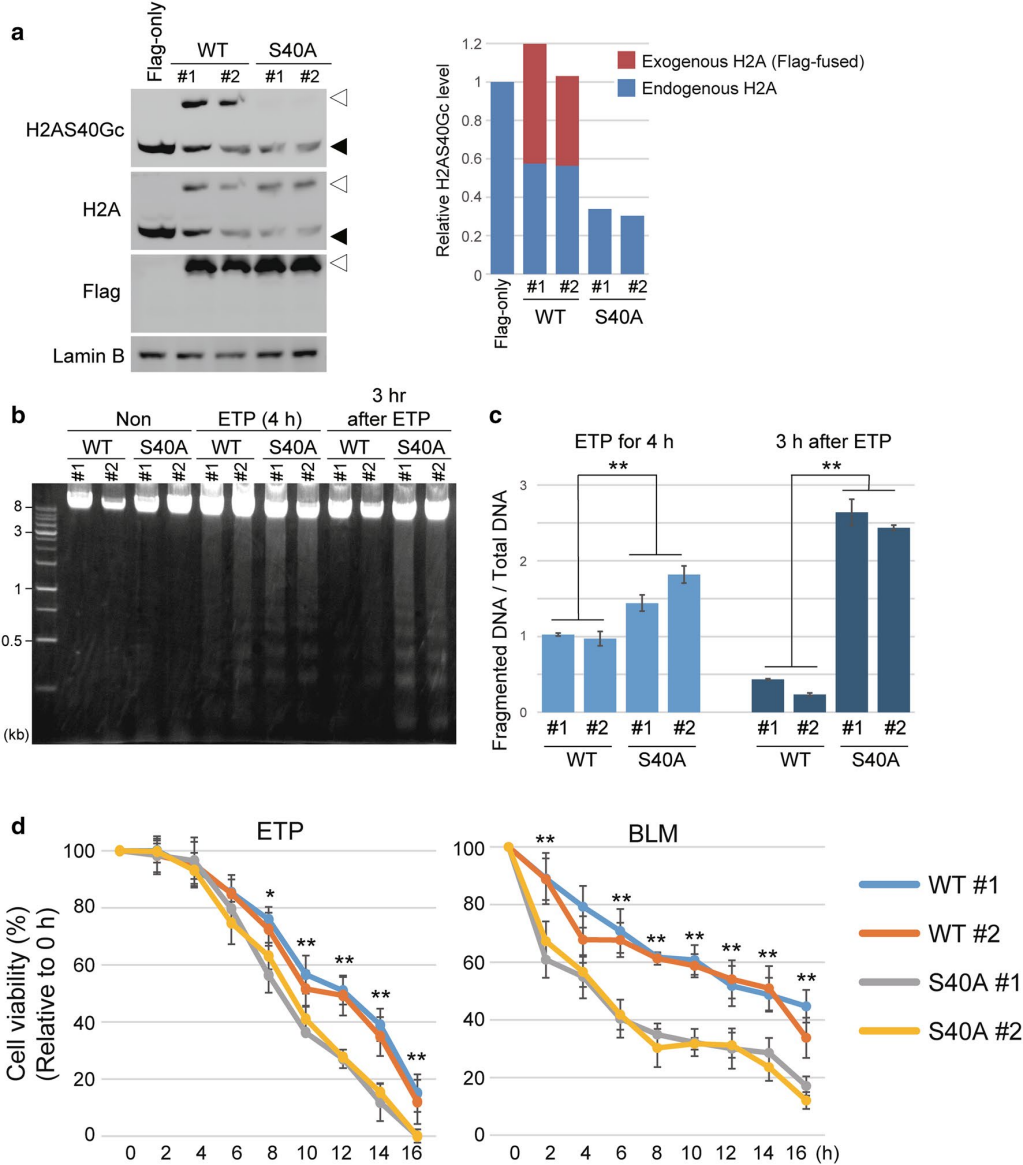
H2AS40Gc is involved in the DNA damage repair mechanism. **a** Left, WB analysis in mESC lines stably expressing 3 × FLAG-fused H2A3-WT (WT) or S40A mutant. Lamin B was used as the internal control. Right, bar graph was visualized from the band intensities of endogenous (black arrowhead) and exogenous (white arrowhead) H2AS40Gc calculated by the ImageJ software. Values were normalized by the intensities of Lamin B and expressed as relative to expression of endogenous H2AS40Gc in mESCs expressing Flag-only. **b** Electrophoresis image of genomic DNA of H2A3-WT mESCs (WT) and S40A mESCs (S40A-mutant) treated with ETP. **c** Ratio of the fragmented genomic DNA in H2A3-WT and S40A-mutant mESCs treated with ETP. Bar graph was visualized from intensities of electrophoresis image calculated by the ImageJ software. Values of fragmented genomic DNA were normalized to the intensity of total genomic DNA and expressed as the relative ratio in biological triplicate experiments. **p < 0.01 (Student’s *t*-test). **d** Time-course experiments of the viability of mESCs in the presence of ETP and bleomycin (BLM). mESCs stably expressing H2A3-WT and S40A-mutant were used, and cell viabilities were measured by the WST-1 assay. Values were normalized to 0 h and indicated as mean ± S.D. (*n* = 3). **p < 0.01, *p < 0.05 (Student’s *t*-test, WT #1 & #2 versus S40A #1 & #2)

To verify the hypothesis of H2AS40Gc functioning in the maintenance of genome integrity, we prepared mESCs stably overexpressing either 3 × FLAG-tagged wild-type H2A3 (H2A3-WT mESC; two lines #1 and #2) or 3 × FLAG-tagged mutant in which serine at position 40 was substituted with alanine (S40A-mutant mESCs; two lines #1 and #2; Fig. 4). Flag-fused H2A3 could be *O*-GlcNAcylated in H2A3-WT mESCs, whereas no *O*-GlcNAcylation was observed in S40A-mutant mESCs (Fig. 4a, *left*). *O*-GlcNAc level of endogenous H2A was reduced to approximately 60 and 30% in H2A3-WT and S40A-mutant mESCs, respectively, compared to that in control mESCs expressing Flag-only (Fig. 4a, *right*). Consequently, total level of *O*-GlcNAcylated H2AS40 consisting of endogenous H2A and the exogenous Flag-H2A3 was much lower in the S40A-mutant mESCs than those in H2A3-WT mESCs (Fig. 4a, *right*). Thus, the set of H2A3-WT and S40A-mutant mESCs can be used for mechanistic analysis of H2AS40Gc.

Therefore, we evaluated the genome damage by treatment with ETP (Fig. 4b) in H2A3-WT and S40A-mutant mESCs. In these cells, ETP treatment for 4 h caused fragmentation of DNA. The level of DNA fragmentation was significantly less in the H2A3-WT mESCs compared to that in S40A-mutant mESCs (Fig. 4b, c), suggesting that H2AS40Gc functions to protect the genome against DNA damage. Then, we checked the cell viability of the WT- and S40A-mutant mESCs in the presence of ETP or bleomycin (BLM), a genotoxic anticancer reagent (Fig. 4d). H2A3-WT mESCs showed more resistance to the genotoxic reagents compared to the S40A-mutant mESCs.

We also investigated genome recovery after ETP-induced DNA damages (Fig. 4b, c). In H2A3-WT mESCs, fragmented DNA reduced evidently 3 h after cessation of ETP treatment. In contrast, DNA fragmentation advanced further in the S40A-mutant mESCs, suggesting that H2AS40Gc has a role in DDR.

### Accumulation of DNA-PKcs and Rad51 requires H2AS40Gc in DDR

Various DDR-related proteins have been identified that play critical roles in the DDR process [33, 34]. For example, DNA-PKcs works at the first step of the homologous recombination process and Rad51 works at the later step of non-homologous end-joining process. ETP treatment for 4 h caused an increase in DNA-PKcs and Rad51 in mESCs (Additional file 1: Figure S1). The overlapping of H2AS40Gc foci with DNA-PKcs and Rad51 foci increased to 15.5 and 25.4% by ETP treatment, respectively (Additional file 1: Figure S1).

**Fig. 5:**
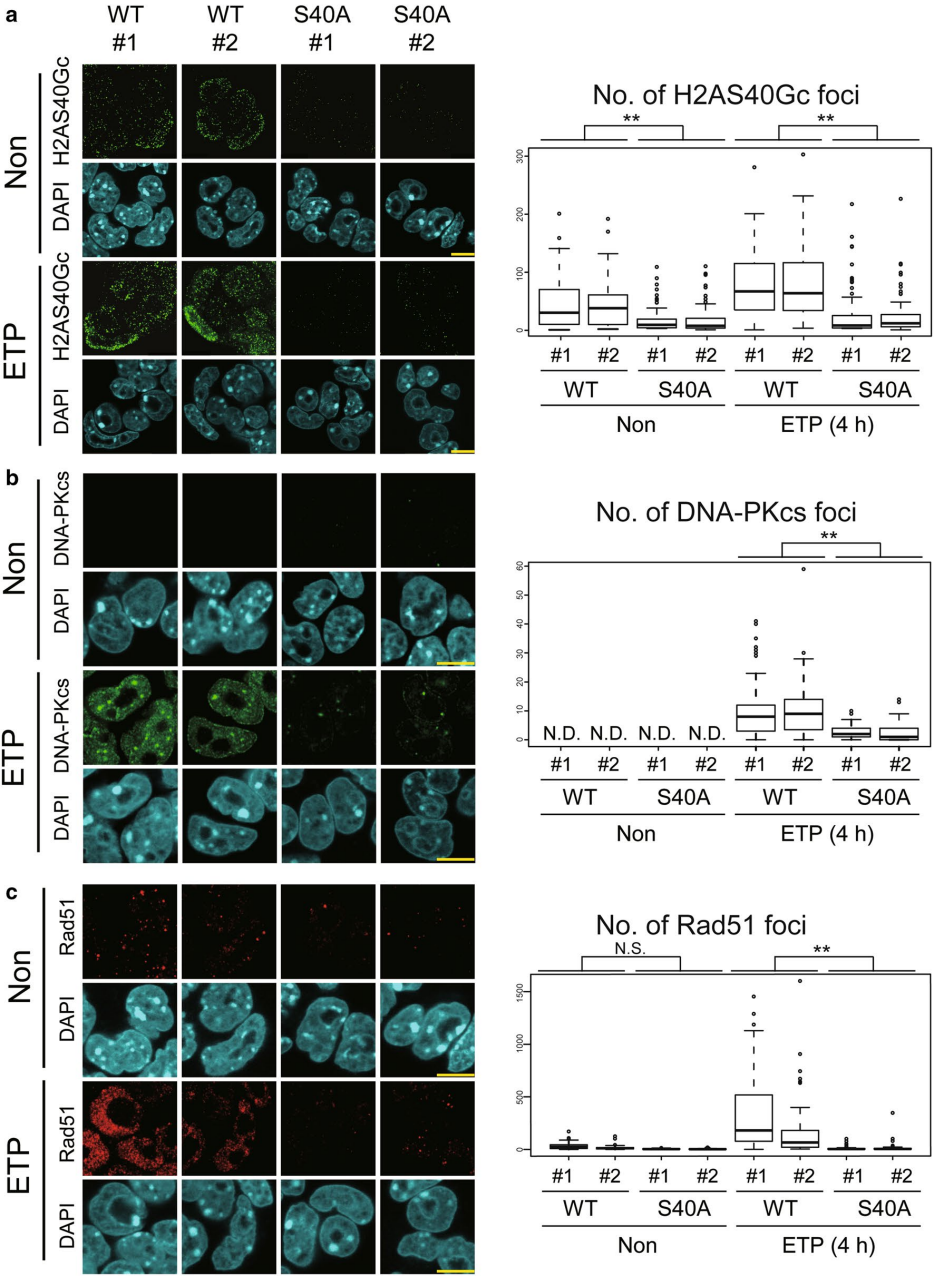
Elevation of H2AS40Gc is important for nuclear accumulation of DNA damage response proteins.** a**–**c** IF images (left) and the number of foci of H2AS40Gc (**a**), DNA-PKcs (**b**), and Rad51 (**c**) (right) in H2A3-WT and S40A-mutant mESCs. **p < 0.01

To get insights into the mechanism of DDR by H2AS40Gc, we next investigated whether DNA-PKcs and Rad51 were induced by ETP in S40A-mESCs. The elevation in H2AS40Gc foci with ETP treatment could be confirmed in H2A3-WT mESCs (Fig. 5a). This change reflected the total S40 *O*-GlcNAcylation of endogenous H2A and exogenous Flag-H2A3 (Fig. 4a). In the S40A-mutant mESCs, the number of H2AS40Gc foci was quite low in the non-treated condition and did not increase by ETP treatment (Fig. 5a). ETP treatment caused an increase in the dotted foci of DNA-PKcs and Rad51 in H2A3-WT mESCs. Importantly, such an increase in DNA-PKcs and Rad51 foci by ETP treatment was not observed in the S40A-mutant mESCs (Fig. 5b, c).

In western blotting analysis, γH2AX increased similarly in both H2A3-WT and S40A-mutant mESCs in response to ETP treatment, and the level was maintained 3 h after the recovery period (Additional file 1: Figure S2). The level of H2AX was constant with or without ETP in both H2A3-WT and S40A-mutant cells, suggesting that phosphorylation at S139 of H2AX was not affected by the decreasing *O*-GlcNAcylation of S40 of H2A. Slight increase in AcH2AZ was observed in S40A-mutant mESCs in response to ETP treatment.

Taken together, these data suggest that H2AS40 is required for the induction of DDR factors such as DNA-PKcs and Rad51 without affecting phosphorylation of H2AX.

### H2AS40Gc associates with AcH2AZ and γH2AX during DDR process

**Fig. 6:**
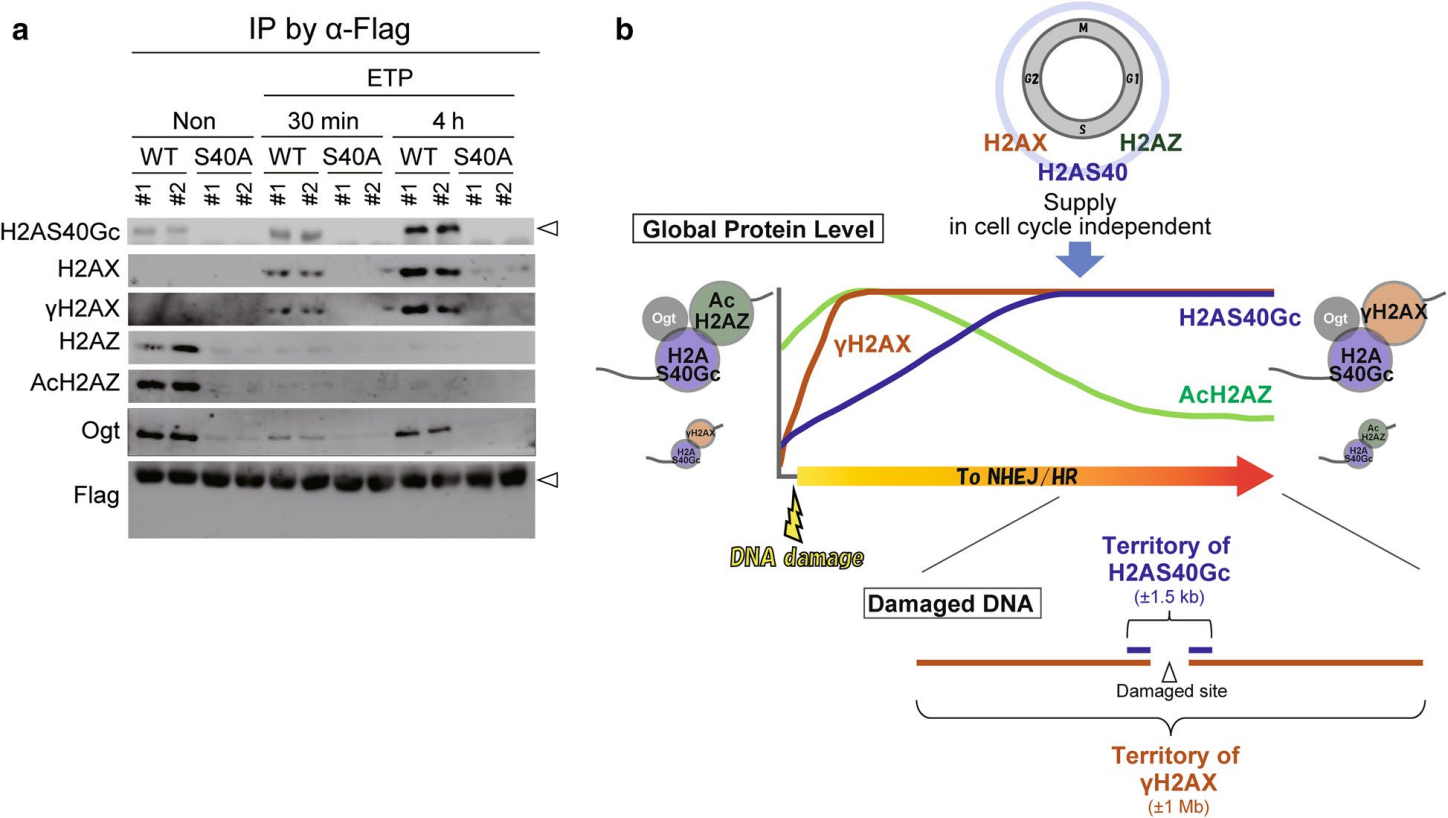
H2AS40Gc partner changes from AcH2AZ to γH2AX during DNA damage response. **a** Binding of H2AS40Gc with H2A variants and Ogt in H2A3-WT and S40A-mutant mESCs during ETP treatment. These binding states were analyzed by immunoprecipitation assay using anti-Flag antibody. White arrowheads indicate exogenous H2A. Input data are shown in Additional file 1: Figure S3. **b** Perspective model for the dynamics of H2AS40Gc during DNA damage response

To determine the direct association of H2AS40Gc with AcH2AZ or γH2AX, immunoprecipitation (IP) using anti-FLAG antibody was performed with nuclear fractions of H2A3-WT and S40A-mutant mESCs (Fig. 6a and Additional file 1: Figure S3). *O*-GlcNAcylation on H2AS40 was confirmed in the IP samples of H2A3-WT, but not in S40A mutants, and increased by ETP treatment in H2A3-WT mESCs. In the non-treated cells, AcH2AZ, but not γH2AX, co-precipitated with H2A3-WT (Fig. 6a). At 30 min after ETP treatment, however, the co-precipitation of AcH2AZ became undetectable. In contrast, γH2AX started to be co-precipitated with H2A3-WT. Co-precipitation of γH2AX with H2A3-WT further increased at 4 h after the treatment. No association of the S40A mutant with AcH2AZ or γH2AX was detected.

*O*-GlcNAc transferase (Ogt) is the sole enzyme known to catalyze *O*-GlcNAcylation [3–5, 35]. It is interesting to note that Ogt was co-precipitated only with H2A3-WT. The associated Ogt decreased at 30 min and then increased again at 4 h after treatment with ETP. These data indicate the physiological interaction among the H2AS40Gc, AcH2AZ, γH2AX, and Ogt during the DDR process. Importantly, the association of H2AS40Gc with AcH2AZ shifts to the association with γH2AX by the advancing DDR mechanism.

## Discussion

In this study, the critical finding was that H2AS40Gc accumulated at the restricted narrow area of DNA damage sites, where H2AS40Gc associated with AcH2AZ and then associated with γH2AX during the DDR process. Compared to H2A3-WT mESCs, S40A-mutant mESCs with lower H2AS40Gc were less resistant to chemical-induced DNA damage. Furthermore, H2AS40Gc was required for accumulation of DDR factors such as DNA-PKcs and Rad51 at the damage site.

A40 to S40 in H2A is a rare mutation in mammals to produce diversity in the nucleosome structure [13]. We found several similarities between S40-type H2A and histone H2A variants: (*i*) S40-type H2A isoforms susceptible for *O*-GlcNAcylation are produced throughout the cell cycle; (*ii*) among the S40-type H2A isoforms, *Hist3h2a* (encoding H2A3) is particularly located as a single gene at the histone gene cluster 3; and (*iii*) the mRNA of S40-type H2A isoforms has poly(A) tail structure. Therefore, S40-type H2A isoforms, including H2A3, are equipped with characteristics similar to those of histone variants such as H2AX and H2AZ. Thus, at least, H2A3 may be considered as a histone variant.

De-condensation of chromatin is required to improve the accessibility of the nucleosomal DNA to the transcriptional and DDR apparatus [36–40]. Nucleosomes containing histone variants lead to profound chromatin alteration and thus influence a multitude of biological processes ranging from transcriptional regulation to genome stability [15, 16, 21, 22]. Evolutionary changes in A40-type H2A prototype into S40-type and consequent *O*-GlcNAcylation may contribute to the variation in the nucleosome [13]. The findings of the present study indicate that H2AS40Gc, in collaboration with AcH2AZ and γH2AX, works as a component of the DDR apparatus. ChIP-seq analysis of mouse trophoblast stem cells revealed that H2AS40Gc is enriched at the genic loci [13]. In the present study, we induced DNA damage at sites in the genic and intergenic area by using CRISPR/CAS9 and found that the territory around H2AS40Gc including both genic and intergenic areas is involved in the response to genome damage. Thus, in addition to regulation of gene expression, H2AS40Gc might maintain genome integrity.

In the initial phase of the DDR process, the incorporation of H2AZ into chromatin and its subsequent rapid removal create an open nucleosome structure [28, 29], and followed by the continued presence of H2AX in the chromatin [26, 27]. Previous reports have shown that nucleosomes containing H2AZ are less stable and that H2AZ exchange promotes the formation of open, relaxed chromatin in response to DNA damages [28, 29]. In the present study, S40-type H2A3 was co-precipitated with AcH2AZ before ETP treatment. Structural studies indicate that steric clashes preclude hetero-dimerization of H2A and H2AZ in the same nucleosome [16, 21, 32]. Because the interaction between the two H2A proteins in the nucleosome occurs via their L1 domains, H2AS40Gc may be involved in the formation of less stable nucleosomes with AcH2AZ at DNA damage sites.

In the case of γH2AX, phosphorylation is required for incorporation into the nucleosome at the DNA damage sites [40, 41], whereas H2AZ is acetylated after incorporation into the nucleosome [42, 43]. Therefore, these posttranslational modifications of histones occur before and after incorporation into the nucleosome, depending on the histone modification. Considering the decrease in the association of Ogt with H2A3-WT at the early phase of ETP treatment, H2AS40Gc might be incorporated into the nucleosome after *O*-GlcNAcylation outside the nucleosome. However, we cannot exclude the possibility of H2AS40 *O*-GlcNAcylation in nucleosome because Ogt co-localized with H2AS40-WT before ETP treatment. Moreover, the timing and mechanism of *O*-GlcNAcylation remains to be investigated.

To date, several *O*-GlcNAcylated sites on the serine or threonine of core histones have been reported [6–13]. Recently, *O*-GlcNAcylation on H2AX at Ser139 (S139) was found in DNA damage foci [44]. It is well established that γH2AX expands to a mega-base region from the site of DNA damage before completion of the repair process [24, 27]. Interestingly, the *O*-GlcNAcylation of H2AX is induced by γH2AX and seems to act as a negative feedback regulator to limit the expansion of γH2AX at the site of DNA damage. S139 on H2AX is the phosphorylation site by ATM/ATR/DNA-PKcs [45–48], and competition between *O*-GlcNAcylation and phosphorylation of H2AX has been postulated [44]. Considering the competition theory, H2AS40Gc may affect the level of phosphorylation [3]. In this case, lack of this phosphorylation event caused by *O*-GlcNAcylation may also contribute to the observed phenotype. Alternatively, overexpression of H2A3-WT may also increase the level of S40-phosphorylated H2AS (H2AS40P). Unfortunately, however, the phosphorylation at S40 of H2AS40-type isoforms has not been identified, and an antibody specific for H2AS40P has not yet been developed. Taken together with the present finding that H2AS40Gc is induced by DNA damage, accumulates in the restricted area (~ 1.5 kb) in the vicinity of the induced site of DNA damage, and interacts with AcH2AZ and γH2AX during the DDR, we conclude that H2AS40Gc plays a role in maintaining genome integrity along with AcH2AZ and γH2AX.

## Conclusions

Here, we propose a model for H2AS40Gc function in the DDR mechanism (Fig. 6b). H2AS40Gc is supplied in response to DNA damages by constant production of S40-type H2A and subsequent *O*-GlcNAcylation occurs. H2AS40Gc, AcH2AZ, and γH2AX are recruited at the DNA damage site. After the occurrence of chemical-induced DNA damage, H2AS40Gc interacts with AcH2AZ only at the initial phase of DDR process, and after that, H2AS40Gc is bound to γH2AX. Thus, the nucleosome members at the DNA damage site change from H2AS40Gc/AcH2AZ to H2AS40Gc/γH2AX during the repair process. Occupancy of γH2AX extends to mega-base limit along the chromatin during the repair process, whereas H2AS40Gc resides in the restricted area. This accumulation of H2AS40Gc seems to be required to form the foci of DNA repair apparatus such as DNA-PKcs and Rad51. In conclusion, S40-type H2A such as H2A3 shows characteristics similar to the histone variants rather than canonical H2A. The present study suggested that H2AS40Gc functions to maintain the genome integrity through DDR mechanism in association with AcH2AZ and γH2AX.

## Methods

### Reagents

Reagents were purchased from Wako Pure Chemicals unless otherwise stated. Camptothecin (CPT), etoposide (ETP), and bleomycin (BLM) (Cayman Chemical) were dissolved in DMSO at concentrations of 20 mM, 100 mM, and 10 mg/mL, respectively, to make stock solutions and stored at − 20 °C. Primers were purchased from Sigma-Aldrich, Eurofins Genomics, or FASMAC and are listed in Additional file 1: Table S1. Antibodies used in this study are listed in Additional file 1: Table S2.

### Cell culture

The mouse embryonic stem cell (mESCs) line J1, derived from 129S4/SvJae mouse embryos, was kindly provided by Dr. En Li [49]. The mESCs were cultured on a gelatin-coated dish (Sigma-Aldrich) in D-MEM (high glucose) supplemented with 15% FBS, 0.1 mM β-mercaptoethanol, 2 mM l-glutamine, 1 mM Na pyruvate, non-essential amino acids, and 1500 U/mL LIF (ESGRO; Millipore). For induction of DNA damages, mESCs were cultured in the presence of CPT (50 µM) or ETP (100 µM) for up to 4 h. Synchronization of mESCs was performed as previously reported [50].

### Cell viability assay

The cell viability after the treatment of ETP and BLM was determined using the cell proliferation reagent WST-1 (Roche). mESCs were plated at 1 × 10^4^ cells/well in 96-well plates in 100 μL of culture medium and cultured for 24 h before use. The medium was changed to new ones containing ETP (100 μM) and BLM (20 μg/mL), and mESCs were incubated at 37 °C for 2–16 h. At the end of the culture, 10 μL of WST-1 was added to each well, and after an incubation for 2 h at 37 °C, the absorption of each well at 440–655 nm was measured using a plate reader iMark (Bio-Rad).

### Construction of overexpression vectors

The pENTR/D-TOPO vector-cloned 3 × FLAG-only, 3 × FLAG-fused H2A3-WT, and its serine 40 to alanine 40 mutant (S40A) were subcloned into pCAG-DEST-IRES-Puromycin-P2A-Venus-pA vector [2, 13] using Gateway LR Clonase (Invitrogen). For induction of site-specific DNA damage using CRISPR/CAS9, assembly of gRNA cassettes into pX458 vector (Addgene) was performed using the Golden Gate cloning method as described previously [51]. For construction of nuclease-deficient CAS9, D10 and H840 of CAS9 were mutated into alanine by PCR amplification. The sequences of resulting constructs were confirmed by BigDye sequencing (Applied Biosystems).

### Transfection

mESCs were cultured in six-well plates to ~ 50% confluence and transfected with 2 μg of plasmid DNA using 4 μL of jetPRIME (Polyplus) per well. For the establishment of mESCs stably expressing 3 × FLAG-H2A3, cells were replated onto a 10-cm dish at 24 h after transfection and cultured for a week in the presence of 10 μg/mL puromycin. Puromycin-resistant colonies were transferred individually to a 96-well plate. Cells that expressed the fusion proteins were expanded, collected, frozen in liquid nitrogen, and stored in cell cryopreservation medium (CELLBANKER 1, Takara Bio) at − 80 °C.

### Chromatin immunoprecipitation (ChIP) assay

ChIP assay was performed using ChIP-IT Express Kit (Active Motif) according to the manufacturer’s instructions with minor modifications. Briefly, the fixed cells were lysed and chromatin was sheared using an enzymatic shearing cocktail for 10 min at 37 °C. The sheared chromatin was mixed with 3 μg of antibody and 40 μL of magnetic beads (Dynabeads M-280 sheep anti-mouse IgG or Dynabeads ProteinG (Invitrogen)) and incubated with rotation at 4 °C overnight. After IP, DNA was recovered by incubation with elution buffer (10% SDS, 300 mM NaCl, 10 mM Tris–HCl, and 5 mM EDTA, pH 8.0) at 65 °C for 6 h. Recovered DNA was purified using ChIP DNA Clean and Concentration Kit (Zymo Research) and was subjected to ChIP-quantitative PCR (qPCR).

### RNA extraction, cDNA synthesis, and genomic DNA extraction

Total RNA was isolated from cells using Direct-zol RNA Kit (Zymo Research) according to the manufacturer’s instructions. First-strand cDNA was synthesized from 1 μg of total RNA using SuperScript III (Invitrogen) and oligo(dT)_20_ primer (in Fig. 1d, *middle*), random hexamer (in Fig. 1d, *right*), or a mixture of both (in Fig. 1d, *left*). Genomic DNA was extracted from mESCs treated with CPT or ETP as described previously [52]. To investigate fragmentation status, genomic DNA was subjected to electrophoresis on 1.2% agarose gel and stained using GelRed (Biotium).

### Quantitative PCR

ChIP-qPCR was performed with KOD SYBR qPCR Mix (Toyobo) using Light Cycler 96 (Roche). PCR was performed with the following thermocycling conditions: denaturation at 95 °C for 1 min and 40 cycles of denaturation at 95 °C for 10 s and elongation at 60 °C for 30 s. For site-specific DNA damage assay by CRISPR/CAS9, ChIP signals were calculated as ChIP DNA/Input DNA and expressed as fold enrichment relative to the uncut (mESCs expressing guide RNA and nuclease-deficient CAS9).

Gene expression analyses of histone coding genes were performed using a high-throughput gene expression platform based on microfluidic dynamic arrays (BioMark, Fluidigm) as described previously [52]. Results of RT-qPCR of histone genes by BioMark were visualized as a heatmap using MeV software [53]. Statistical analysis was performed using Student’s *t* test.

### Immunofluorescence analysis

Cells cultured in four-well plates were fixed with 4% paraformaldehyde and permeabilized with 0.2% Triton X-100 followed by blocking with 5% BSA (Rockland)–Tween 20–PBS and incubation with the primary antibody overnight at 4 °C. The secondary antibody was added, and incubation was continued for another 1 h at room temperature. Nuclei were stained with DAPI (1 μg/mL; Dojindo). Fluorescence images were acquired with a confocal microscope FV10i (OLYMPUS). For measurements of foci of H2AS40Gc and DDR-related proteins, merged immunofluorescence images of DAPI and the proteins were processed using CellProfiler software [54]. At least 50 nuclei were used for the measurements per protein. Statistical analysis was performed by Wilcoxon rank sum test.

### Western blotting

Nuclear proteins of each sample were collected using LysoPure Nuclear and a Cytoplasmic Extractor Kit according to the manufacturer’s protocols. Proteins were fractionated by 20% SDS–PAGE (XV PANTERA Gel; DRC), blotted onto PVDF membranes (Immobilon-P, Millipore), and blocked with 5% skim milk—0.1% Tween 20–TBS for 1 h and incubated at 4 °C overnight with the primary antibody diluted in 1% BSA–0.1% Tween 20–TBS. For detection of H2AS40Gc by 20B2, the blotted membrane was blocked with 5% BSA–0.1% Tween 20–PBS (blocking buffer) and washed with 0.1% Tween 20–PBS, and antibody was diluted using blocking buffer. Protein bands were detected using secondary antibody conjugated with horseradish peroxidase (Jackson ImmunoResearch), and ImmunoStar Basic or ImmunoStar LD.

### Immunoprecipitation

For the immunoprecipitation of the 3 × FLAG-tagged H2A isoforms, nuclear fractions (100 μg each) were mixed with 20 μL anti-FLAG M2 Magnetic Beads (Sigma-Aldrich) and incubated with rotation at 4 °C overnight. Precipitates were washed five times with TBS, and the beads were suspended in Lane Marker Reducing Sample Buffer (Thermo) and boiled to extract proteins.

## Additional file

**Additional file 1:** Figure S1-S3 and Table S1-S2.

## Abbreviations

*O*-GlcNAc: *O*-linked β-*N*-acetylglucosamine
H2AS40Gc: *O*-GlcNAc modification at serine 40 of H2A
mESC: mouse embryonic stem cell
DDR: DNA damage repair
Ogt: *O*-GlcNAc transferase
CPT: camptothecin
ETP: etoposide
ChIP: chromatin immunoprecipitation
