# Supplementary material for: H2A O-GlcNAcylation at serine 40 functions genomic protection in association with acetylated H2AZ or γH2AX
Source: Epigenetics Chromatin. 2017 Oct 30;10:51. doi: 10.1186/s13072-017-0157-x (PMC5663087; doi:10.1186/s13072-017-0157-x)
Supplement: Supplementary file 1 — Additional file 1: Figure S1-S3 and Table S1-S2. [file 13072_2017_157_MOESM1_ESM.doc]

**Supplementary Material**

**H2A *O*-GlcNAcylation at serine 40 functions genomic protection in association with acetylated H2AZ or γH2AX**

Koji Hayakawa^1^*, Mitsuko Hirosawa^1^*, Ruiko Tani^1^, Chikako Yoneda^1^, Satoshi Tanaka^1^,

Kunio Shiota^1,2^**

^1^, Laboratory of Cellular Biochemistry, Department of Animal Resource Sciences/Veterinary Medical Sciences, The University of Tokyo, Tokyo 113-8657, Japan

^2^, Waseda Research Institute for Science and Engineering, Waseda University, Tokyo 169-8555, Japan

*, These authors contributed equally to this work

**, To whom correspondence should be addressed: Kunio Shiota. Laboratory of Cellular Biochemistry, Department of Animal Resource Sciences/ Veterinary Medical Sciences, The University of Tokyo, Tokyo 113-8657, Japan

TEL/FAX: +81-3-5841-5472/+81-3-5841-8189

Email: ashiota@mail.ecc.u-tokyo.ac.jp or ashiota@aoni.waseda.jp

**Figure S1-S3**

**Table S1-S2**

**
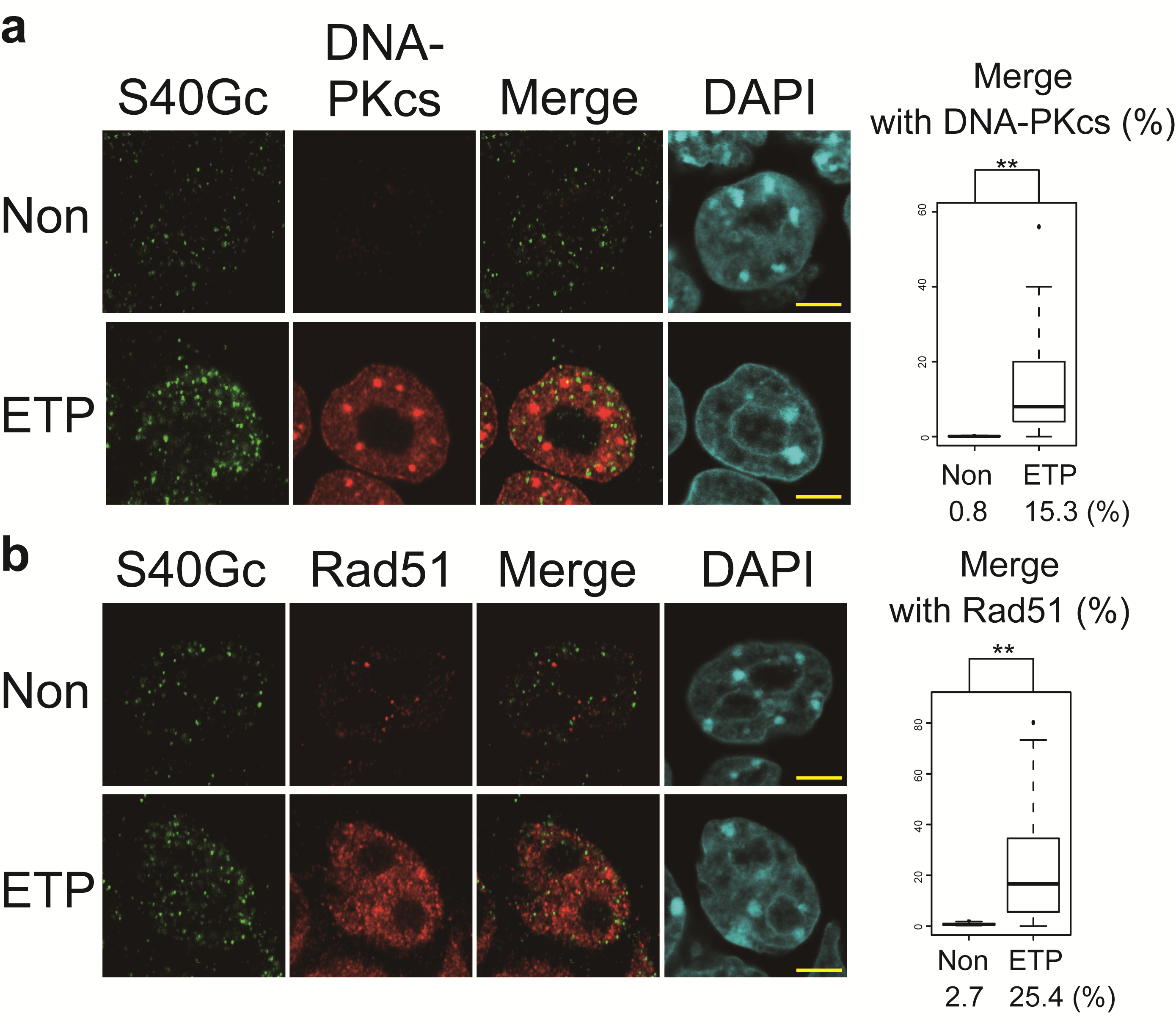
**

**Figure S1** Co-localization of H2AS40Gc with DNA-PKcs and Rad51 in mESCs

**a and b** *left*, IF images of H2AS40Gc, DNA-PKcs (**a**), and Rad51 (**b**) in mESCs treated with ETP. Bars = 5 μm. *Right*, measurement of co-localization of H2AS40Gc with DNA-PKcs (**a**) or Rad51 (**b**) in nuclei of mESCs treated with ETP. ** *P* < 0.01.


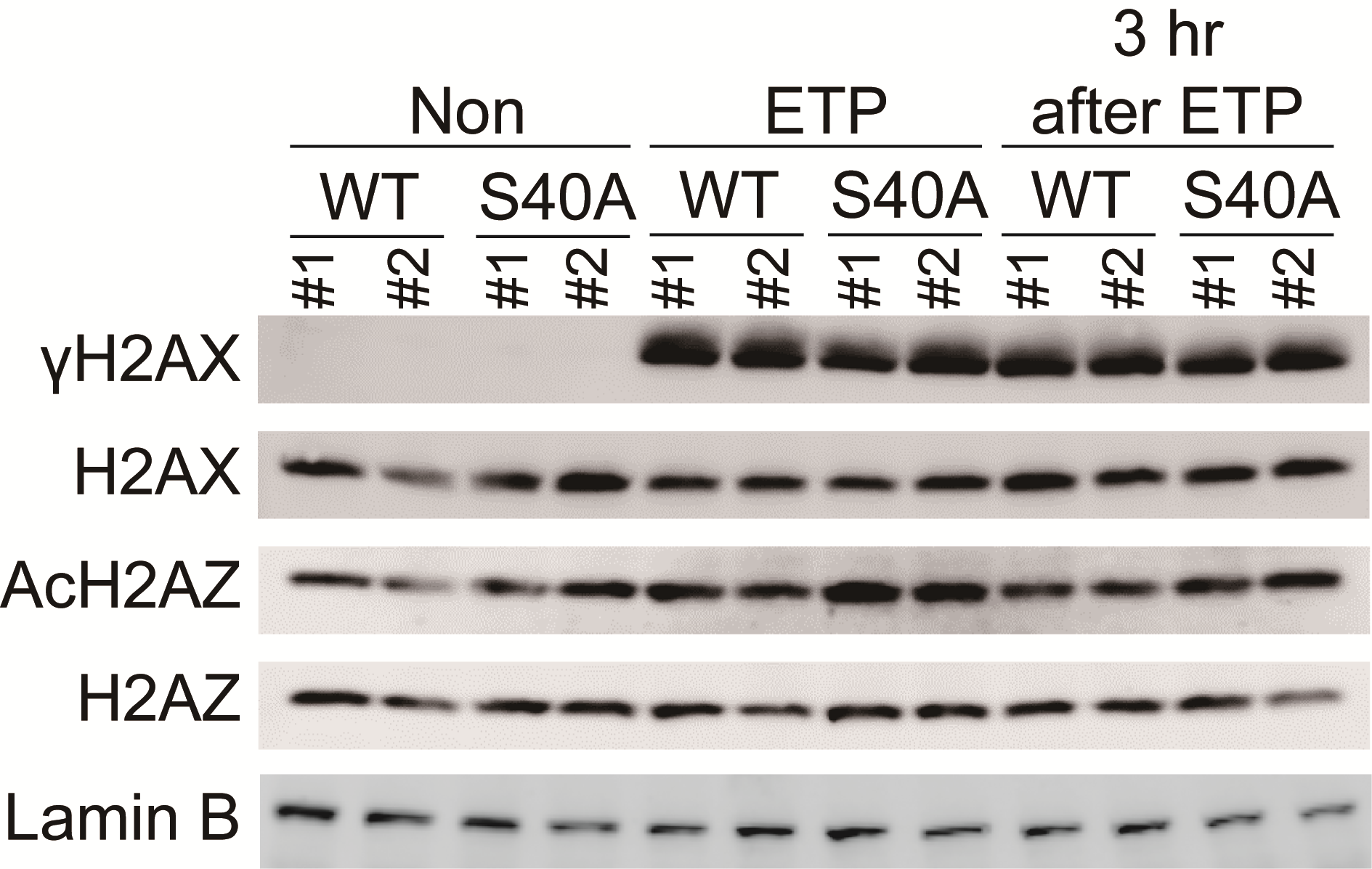


**Figure S2** WB analysis of H2AX and H2AZ in H2A3-WT and S40A-mutant mESCs treated by ETP.

Lamin B was used as an internal control.

**
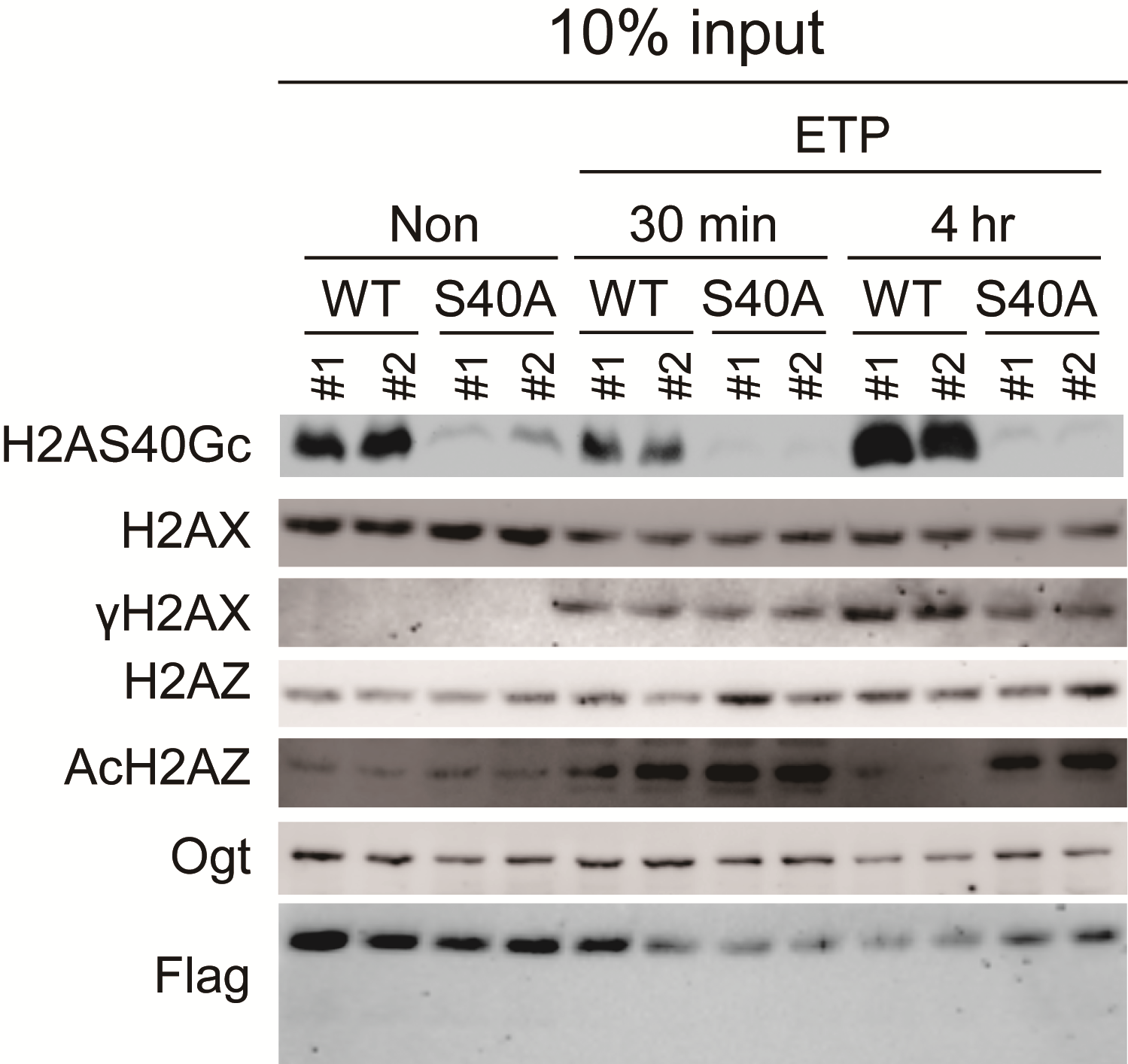
**

**Figure S3** Levels of H2AS40Gc, γH2AX, AcH2AZ and O-GlcNAc transferase (Ogt) used in immunoprecipitation experiments depicted in figure 6a.

**Table S1. Primer list**

**For RT-qPCR using BioMark system***

| Primer Name | Forward | Reverse | Universal probe ID |
| --- | --- | --- | --- |
| Hist1h2aa | GTATCTGGCGGCAGTGCTA | TAATGCGCGTCTTCTTGTTG | #1 |
| Hist1h2ab | GTCCTGCCCAACATCCAG | CAGTACAGACAATCGCCAGATTA | #74 |
| Hist1h2ad | GTCCTGCCCAACATCCAG | TTATTTCCCCTTGGCCTTG | #74 |
| Hist1h2af | GTCCTGCCCAACATCCAG | CTTTCCCTTGGGCTTATGG | #74 |
| Hist1h2ag | GTCCTGCCCAACATCCAG | CAACAGTGCTTTGTATAAAGGGTTT | #74 |
| Hist1h2ah | GTCTGGACGCGGTAAGCA | CCGAGTAGTTGCCCTTGC | #52 |
| Hist1h2ak | TGGCAGCCGTGCTAGAGTA | TAATGCGCGTCTTCTTGTTG | #1 |
| Hist1h2an | ACGACGAGGAGCTCAACAA | CTTCTTCGGCAGCAGTACG | #107 |
| Hist1h2ao | GTCCTGCCCAACATCCAG | TTTTCCCTTGGCCTTGTG | #74 |
| Hist2h2aa1 | GCCCGCGTCTCTGTGATA | AGACCGGCTACCGTGACA | #21 |
| Hist2h2aa2 | GTCCTGCCCAACATCCAG | CTTGCCCTTCGCCTTATG | #74 |
| Hist2h2ab | CGGTGCTGGAGTACCTAACG | TTCCTCACAGCTAGTTGCAGAT | #1 |
| Hist2h2ac | AAAGTGACGATCGCACAGG | CTTGTGGCTCTCGGTCTTCT | #74 |
| Hist3h2a | TGGAGGGAGGTGTACTAGGG | TTGGGTAGTGGTTGTGCATTT | #66 |
| H2afx | AAGCCGGTGAATCCCTGT | AGCTGCAAAAGTTCCAGTTCA | #106 |
| H2afz | CTCAGGACTCTAAATATTCCTAACAGC | TGTTTTTCACAGAGATACAGTCCAC | #47 |
| H2afy | TGACATTGACCTTAAAGATGACCT | CCAGAACAGCTTCTACAAACTCC | #47 |
| H2afy2 | AGTGGGGCTCCGACAAAT | TGCAGACAGGCAGTTTTTGA | #11 |

*, There are 18 canonical histone H2A isoforms, of which thirteen S40 and five A40 types are present in mouse. We investigated the expression profile of fourteen H2A isoforms and four histone variants by RT-qPCR using specific primer sets in mESCs (Fig. 1), because the primer sets for the four genes (*Hist1h2ac*, *Hist1h2ae*, *Hist1h2ai* and *Hist1h2ap*) did not work in the RT-qPCR.

**For construction of gRNA expression vector targeted to chromosome 3 and 13**

| Primer Name | Forward | Reverse |
| --- | --- | --- |
| Chr3_gRNA | CACCGAGAGTTCGGTTGCTTCGGTTGGG | AAACCCCAACCGAAGCAACCGAACTCTC |
| Chr13_gRNA | CACCGTCCAAGTCCAGTTCACTCGCCGG | AAACCCGGCGAGTGAACTGGACTTGGAC |

**For construction of dCAS9 expression vector**

| Primer Name | Forward | Reverse |
| --- | --- | --- |
| dCas9_D10A | GGCCTGGCCATCGGCACCAACTCTGTG | GCCGATGGCCAGGCCGATGCTGTACTT |
| dCas9_H840A | GTGGACGCT ATCGTGCCTCAGAGCTTT | CACGATAGCGTCCACATCGTAGTCGGA |

**For ChIP-qPCR in DSBs assay by CRISPR/CAS**9 system

| Primer Name | Forward | Reverse |
| --- | --- | --- |
| Chr3_+0.5kb | GAGGTAAGCGATGGTTGAGG | GTGCAGAGTACCGCATCTCA |
| Chr3_+1.5kb | AGCAAAGGAACAATGGGATG | TCCAGGATCACACAGGAAGC |
| Chr3_+3.5kb | AGAGGGGTTAGCCAATGTCA | CTTCCCTCTGTGTGTGTGTG |
| Chr3_+10kb | TTAGCGTGCAAATGGATCAG | TGAGCACCATCTGCAGTTTTC |
| Chr3_+50kb | TTTCTTAGGTTCCGCCTCCT | AGCACAATTCTCCATCATGC |
| Chr3_-0.5kb | GAAGATGTTGGTCCCCCATA | GGAGGATTCCAAAGGCTACC |
| Chr3_-1.5kb | GCGGTGACAAGGGGTATCTA | AGCAAGAACAGCAGCAAACA |
| Chr3_-3.5kb | TGGACAAAGAAGCCAAAACC | CCTTGATCCACTTGGACTTG |
| Chr3_-10kb | TTTACTCCCTAGCACCCACA | ATGCTAGTGCCCACAGGACT |
| Chr3_-50kb | GGGTCTTAGAGGCACATGTGATA | GGAGGGTGTTAATGGGAGAGA |
| Chr13_+0.5kb | CATTTGCATACCTGCGTCTG | GGACAGCAAAAGGTGAGTGG |
| Chr13_+1.5kb | TTTGTGTTGGAAACCAGCAG | TGCCATAAAGGTGAGCCAAG |
| Chr13_+3.5kb | CCTTTTTCTGGGTCTTGGTG | GAGACGTTGGTGTGGGAAAG |
| Chr13_+10kb | TCTGAAAGGCTGAGAACATCC | TGCAATTGTCATCTCCAAGG |
| Chr13_+50kb | GCTCTTTGATTGGCCTTTTG | ACAGAGTGAGTTCCAGGACAGC |
| Chr13_-0.5kb | GCCATTTGGCTTACATTTCC | ACGAGGAGCAAGCCAGTAAAC |
| Chr13_-1.5kb | GCAGATCATCCTGGAAAACC | ACAGCTCCCTTGCAAGTCC |
| Chr13_-3.5kb | TTCCAGAGATCCTGAGTTCAA | TTTGCTTGTTTGTTTGCTCA |
| Chr13_-10kb | CCAACTACTGTCAACATCAAGG | TGGCACAGCAGTTGATTGAT |
| Chr13_-50kb | TCTGCAGGGAGTTTCTGTCC | GTCAGGAAAATGACGGTGCT |

**Table S2.** Antibody list

| **1^st^ antibody** | | | |
| --- | --- | --- | --- |
| Name | Company | Cat. No. | Applications^1^ (Final conc.) |
| Flag | Sigma | F1804 | WB (1 μg/mL) |
| H2A | Millipore | 07-146 | WB (1:1000) |
| γH2AX | Millipore | 05-636 | WB (1 μg/mL), ChIP (30 μg/mL) |
| γH2AX | Abcam | ab2893 | IF (1 μg/mL) |
| H2AX | Abcam | ab11175 | WB (1 μg/mL) |
| Acetylated H2AZ | Abcam | ab18262 | WB (1 μg/mL), IF (1 μg/mL) , ChIP (30 μg/mL) |
| H2AZ | Abcam | ab150402 | WB (1 μg/mL) |
| DNA-PKcs | Abcam | ab18192 | IF (1 μg/mL) |
| Rad51 | Cell signaling | 8875 | IF (1 μg/mL) |
| Cyclin E | Santa Cruz | sc-481 | WB (1 μg/mL) |
| Phospho-H3S10 | Abcam | ab14955 | WB (1 μg/mL) |
| Actb | Sigma | A1978 | WB (1 μg/mL) |
| Lamin B | Santa Cruz | sc-6216 | WB (1 μg/mL) |
| H2AS40Gc (20B2) | Specificity in WB, IF and ChIP experiments was confirmed in the previous study [13]. | | WB (1 μg/mL), IF (1 μg/mL), ChIP (30μg/mL) |
| **2^nd^ antibody** | | | |
| Alexa Fluor 488 conjugated Goat anti-Mouse IgG | Invitrogen | A11029 | IF (2 μg/mL) |
| Alexa Fluor 594 conjugated Goat anti-Mouse IgG | Invitrogen | A11037 | IF (2 μg/mL) |
| Alexa Fluor 594 conjugated Donky anti-Sheep IgG | Invitrogen | A11016 | IF (2 μg/mL) |
| Peroxidase-conjugated  Goat anti-Mouse IgG | Jackson ImmunoResearch | 115-036-062 | WB (0.2 μg/mL) |
| Peroxidase-conjugated  Goat anti-Rabbit IgG | Jackson ImmunoResearch | 111-035-003 | WB (0.2 μg/mL) |
| Peroxidase-conjugated  Donky anti-Sheep IgG | Jackson ImmunoResearch | 713-035-003 | WB (0.2 μg/mL) |
